# Clinical Findings Associated with a De Novo Partial Trisomy 10p11.22p15.3 and Monosomy 7p22.3 Detected by Chromosomal Microarray Analysis

**DOI:** 10.1155/2011/131768

**Published:** 2011-12-08

**Authors:** Omid Kohannim, Jane Peredo, Katrina M. Dipple, Fabiola Quintero-Rivera

**Affiliations:** ^1^David Geffen School of Medicine, UCLA, Los Angeles, CA 90095, USA; ^2^Department of Pediatrics, David Geffen School of Medicine, UCLA, Los Angeles, CA 90095, USA; ^3^Department of Human Genetics, David Geffen School of Medicine, UCLA, Los Angeles, CA 90095, USA; ^4^Department of Pathology and Laboratory Medicine, David Geffen School of Medicine, UCLA, 1000 Veteran Avenue 22-26, Los Angeles, CA 90024, USA

## Abstract

We present the case of an 18-month-old boy with dysmorphic facial features, developmental delay, growth retardation, bilateral clubfeet, thrombocytopenia, and strabismus, whose array CGH analysis revealed concurrent *de novo *trisomy 10p11.22p15.3 and monosomy 7p22.3. We describe the patient's clinical presentation, along with his cytogenetic analysis, and we compare the findings to those of similar case reports in the literature. We also perform a bioinformatic analysis in the chromosomal regions of segmental aneuploidy to find genes that could potentially explain the patient's phenotype.

## 1. Introduction

Chromosomal abnormalities are common causes of multiple congenital anomalies. Complete chromosomal trisomies and monosomies of a few chromosomes (such as trisomy 21 and monosomy X) comprise the most common types of chromosomal abnormalities. Partial chromosomal trisomies and monosomies are less common, but are occasionally accounted in individual case reports. In this article, we report the case of a boy with partial trisomy 10p and partial monosomy 7p revealed by comparative genomic hybridization (CGH) analysis. We describe the patient's multiple congenital anomalies, and compare them with those of previously reported cases of either trisomy 10p or monosomy 7p. We also browse the patient's chromosomal regions of aneuploidy to find genes that could potentially give rise to the patient's signs and symptoms.

## 2. Case Presentation

The patient is an 18-month-old male born to a 29-year-old G1P0 Native American mother and a 40-year-old Honduran father at 40 weeks of gestation with several congenital anomalies. Maternal past medical history was significant for a history of a seizure disorder. She had not had a seizure in three years and was no longer on antiseizure medication. She did not take medications during pregnancy. There is no known family history of major developmental disabilities in either side of the family.

At time of birth, the patient's Apgar scores were 6, 7, and 8 at 1, 5, and 10 minutes, respectively, weight of 2995 g (25th percentile), length of 50 cm (50th percentile), and occipital-frontal circumference (OFC) of 35 cm (30th percentile). Dysmorphic features included bitemporal narrowing, a very wide sagittal suture consistent with a bifid cranium with a large sagittal and metopic suture, posteriorly rotated ears, highly arched palate, micrognathia, frontal bossing, and redundant skin on the neck with a low-lying hairline in the posterior neck. Other findings of his physical examination included hirsutism on the back, congenital hip and knee dislocation bilaterally, bilateral clubfeet (status postcasting), sacral dimples, and enlarged nipples. Abnormal labs included thrombocytopenia (platelet count of 49,000 with normal values of 143,000 to 498,000), which spontaneously resolved. Workup for infections including TORCH infections was normal. He has had issues with recurrent otitis media. Imaging tests, head, renal, and sacral ultrasound along with skeletal survey X-rays were normal, and he passed the newborn hearing screening. 

**Figure 1:**
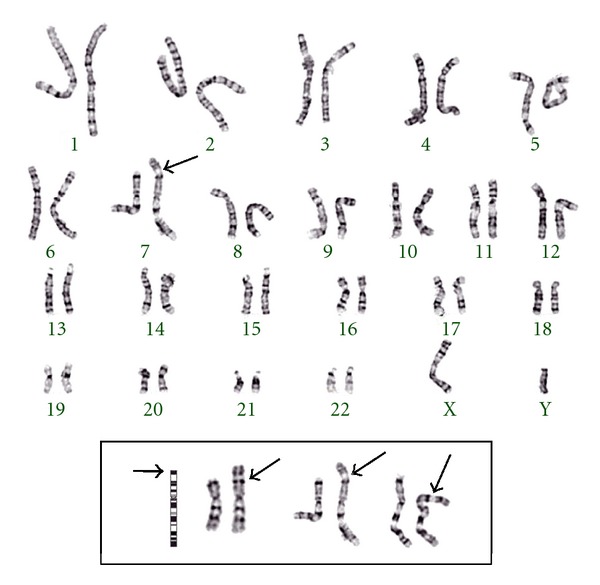
Patient's complete and partial (boxed, different band resolutions) GPG-banded karyograms, displays extraneous chromosomal material at the telomere of chromosome 7p.

**Figure 2:**
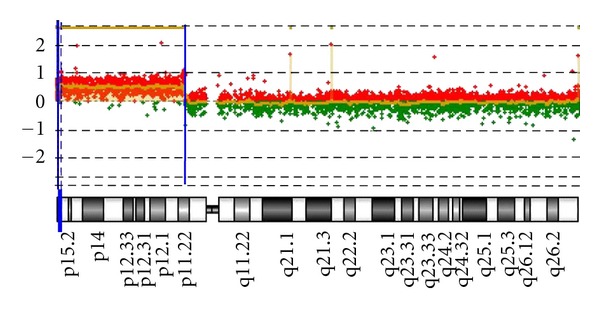
Oligonucleotide-aCGH profile for chromosome 10. The ideogram (lower part) depicts the entire chromosome 10. The 33 Mb gain interval at 10p11.22p15.3 [hg18,chr10: 0-33,408,955 bp] detected in this patient is indicated by two vertical blue bars, and the log_2_ ratio of +1.

Standard chromosome analysis detected an abnormal male karyogram with additional chromosomal material of unidentifiable origin at the end for the short arm of one chromosome 7; 46,XY,add(7)(p22). Additional whole-genome array-based comparative genomic hybridization (aCGH) was performed on the proband and parental peripheral blood samples using the CMDX Oligo HD Scan to determine the origin of the additional material, and its clinical significance. The array contains 99,000+ probes covering coding and noncoding human genome sequences with content sourced from the UCSC hg18 human genome (NCBI build 36, March 2006) and an average probe spatial resolution of ~21 Kb. Complete and partial karyograms are shown in Figure 1. The patient's aCGH analysis identified a copy number loss of approximately 0.5 Mb of the region 7p22.3 and gain of 33 Mb of the short arm of chromosome 10 (Figure 2). The karyotype is denoted as arr 7p22.3(0-749,854)x1,10 p15.3p11.22 (0-33,408,955)x3, 10p11.22(33,039,622-33,408,955)x4 in accordance with the ISCN2009. This result indicates a telomeric deletion of 7p22.3 and partial duplication of 10p11.22p15.3, a result of a *de novo* unbalanced translocation. Parental karyotypes and aCGH were normal. The final revised karyotype is described as 46,XY,der(7)t(7; 10)(p22.3; p11.22).arr 7p22.3(0-749,854)x1,10 p15.3p11.22 (0-33,408,955)x3, 10p11.22(33,039,622-33,408,955)x4 dn.

The patient was evaluated at the genetics clinic for followup when he was 4.5 months old. On physical examination, the patient's weight was 6.2 kg (10th to 25th percentile), height was 66 cm (75th percentile), and OFC was 41.5 cm (25th percentile). In general, he looked well-nourished and in no acute distress. At this time, the patient was also noted to have hypertelorism with strabismus. His developmental milestones were delayed. He had some head control, and was starting to spend time with his face down, playing with his hands, cooing, making noises, and taking his hands to the midline grabbing at objects.

On followup evaluation at 13.5 months of age, he was found to still have developmental delay. The patient had been receiving visual therapy for his strabismus (specifically, esotropia), which was noted to be improving, and he was followed regularly in an ophthalmology clinic. He was also monitored routinely by orthopedics for his lower extremity anomalies, for which he had underwent postnatal, corrective surgeries.

At the most recent genetics evaluation, he was 27 months of age and doing well. He had recurrent infections including otitis media, upper respiratory infections, and aspiration pneumonia. He had recently been diagnosed with asthma. He was growing well and gaining weight easily. He continued to have developmental delay and at 27 months was able to commando crawl and say a few words.

## 3. Discussion

Individual cases of partial trisomy 10p11.22p15.3 and monosomy 7p22.3 have been previously reported in the literature. The combination of the two in a single patient, however, has not been reported. The first case of trisomy 10p, due to an unbalanced t(7; 10) (p22; p11) translocation, was that of two siblings described by Schleiermacher et al. 1974 [1], which have the same region of 10p trisomy and monosomy 7p as our patient (see Table 1). However, at the time of this study, the method of detection was only through standard karotyping, which makes the comparison of gene contribution difficult, and therefore the genotype-phenotype correlation has never been studied before. Since then, several investigators have reported tens of cases of trisomy 10p with some similar and other new phenotypic features. We compared the phenotypic findings in our patient with those of other patients with pure trisomy 10p (since our patient's region of trisomy covers almost all of the short arm of chromosome) and monosomy 7p22.3. The results are shown in Table 1. It should be noted that the 0.37 Kb genomic region on 10p11.22, of which four copies were found in our patient, may point to a normal genomic variation that our method has been able to detect, as searches for this variation in the Database of Genomic Variants (http://projects.tcag.ca/variation/) and the Children's Hospital of Philadelphia (CHOP) copy number variation database (http://cnv.chop.edu/) did not yield any overlapping results.

Our patient's signs and symptoms, including his early growth retardation, feet anomaly, low platelet count, wide fontanelle, high-arched palate, micrognathia, and hypertelorism, have been previously associated with trisomy 10p in various case reports [1, 2, 6, 7]. Posterior ear rotation has also been reported to be associated with some cases of monosomy 7p in the past [3]. Our patient's phenotypic findings are therefore mainly due to his trisomy 10p, with the possible exception of his posterior ear rotation, which may have resulted from his 7p microdeletion. In addition to comparing our patient's case to others in the literature, we also searched the Database of Chromosomal Imbalance and Phenotype in Humans Using Ensembl Resources (DECIPHER; http://decipher.sanger.ac.uk/) for chromosomal aberrations in the affected chromosomal locations. Although many of the cases in the database reported nonspecific or no phenotypic information, several (on chromosome 10p, ranging from 145 Kb to 36.8 Mb) mentioned developmental delay as well as micrognathia, hypertelorism, and anomalies in the skin and feet, in agreement with our case.

These types of rearrangements are due to 2; 2 malsegregation during meiosis I, adjacent 1, single segment is the most likely type of malsegregation underlying this patient's unbalanced translocation, because one of the segments or the portions exchanged, on chromosome 7, is smaller (0.5 Mb) compared to the chromosome 10 segment [8]. The 33 Mb region of chromosome 10p, which our patient has gained a single copy of, contains several RefSeq genes (>500), some of which could have potentially played a role in his multiple congenital anomalies. We identified three of these genes (Table 2), whose protein products' functions are known and are possibly related to the patient's clinical presentation, including but not limited to his hematological (*PFKP, TAF3*) and possible hearing (*MYO3A*) signs and symptoms. *PFKP*, encoding the platelet isoform of the key regulatory phosphofructokinase enzyme, and *TAF3*, coding for an important member of the molecular transcription machinery, reported to be necessary for early hematopoiesis [4], are potential candidate genes for our patient's transient thrombocytopenia. *TAF3*'s contribution to skeletal muscle development [5] may also at least partially explain our patient's clubfeet presentation. *OPTN*, though mostly recognized in the literature for its role in the pathophysiology of glaucoma [9, 10], may be a potential candidate gene for our patient's visual symptoms including strabismus, through unknown mechanisms. It should also be noted that it is critical for our patient to be continuously evaluated for his hearing since loss of function mutations in *MYO3A*, a member of the myosin superfamily of genes on chromosome 10p, have been linked to progressive nonsyndromic hearing loss [11].

Investigation of the few genes in the region of monosomy for chromosome 7p also revealed *FAM20C* and platelet-derived growth factor alpha (*PDGFA*), as genes that have potential roles in our patient's developmental delay and bone findings. *FAM20C* has been shown to be important for normal bone development, as individuals with loss of function mutations in the gene suffer from Raine syndrome, a fatal bone disorder [12]. Patients with less severe mutations in *FAM20C *have been reported to have wide fontanelles, as in our patient [13]. *PDGFA* is also a critical gene for development, particularly for oligodendrocytes, as knockout mice have been observed to die soon after birth or as embryos, most probably due to myelin loss from abnormal oligodendrocyte development [14], making it a potential candidate gene for our patient's developmental delay.

The relatively large region of trisomy on chromosome 10p, in addition to monosomy 7p, has caused several congenital anomalies in our patient, which affect almost all of his organ systems. Other examples of concurrent partial trisomy and monosomy of other chromosomes detected by array CGH, prenatally or postnatally, have been previously described in the literature [15]. Although the exact causes of the patient's single findings are unknown, we were able to identify the nature of the chromosomal abnormalities. We also identified six potential candidate genes (*PFKP*, *TAF3*, *OPTN*, *MYO3A*, *FAM20C,* and *PDGFA*), among ~500 genes in the patient's affected chromosomal regions, which can possibly explain several of his developmentally related signs and symptoms. More patients like ours are expected to be identified through the current use of this powerful, genome-wide, high-resolution array technology. The description of the phenotype and the followup of such patients are critical for the understanding of these new conditions and the counseling of new affected patients. Further research is needed to identify the exact mechanisms by which these genes can cause such clinical findings.

## Figures and Tables

**Table 1:** Phenotypic findings in our patient and patients reported in the literature with either pure trisomy 10p11.2p15.3 or monosomy 7p22.

Name/case	Present case	Schleiermacher et al. [1] ^a^	Granata et al. [2]	Schomig-Spingler et al. [3]
Gender	Male	Male+ Female	Mixed	Mixed
Genotype	10p+, 7p−	10p+	10p+	7p−
Growth retardation	+	+	9/9	ND
High-arched palate	+	+	7/9	7/12
Wide fontanelle(s)	+	+	6/9	ND
Hypertelorism	+	ND	5/9	2/12^b^
Micrognathia	+	ND	5/9	ND
Feet anomaly	+	+	4/9	6/12
Thrombocytopenia	+	ND	1/9	ND
Posterior ear rotation	+	+	ND	1/12

Detection method	aCGH	Karyotype	Karyotype	Karyotype

^a^This paper describes the first case of 10p trisomy, with the same breakpoints as in our patient's case.

^b^The patients were reported to have either hyper- or hypotelorism.

ND: not described.

**Table 2:** Potentially causative genes on chromosome 10p11.22p15.3 that may play a role in our patient's phenotypic findings.

Gene Name	OMIM ID	Gene description	Gene product function	Relation to patient's phenotype
*PFKP*	171840	*Homo sapiens* phosphofructokinase, platelet (PFKP), mRNA	Encodes the platelet isoform of phosphofructokinase (PFK), a key regulatory enzyme in glycolysis. This gene is also expressed in fibroblasts.	Thrombocytopenia
*TAF3*	606576	*Homo sapiens* TAF3 RNA polymerase II, TATA box binding protein (TBP)-associated factor, mRNA	A critical member of the highly conserved transcription factor machinery, TAF3 has been found to be involved in early development and hematopoiesis [4] as well as skeletal muscle differentiation [5].	Thrombocytopenia, clubfeet
*MYO3A*	606808	*Homo sapiens*, Myosin IIIA, mRNA	Encodes a member of the myosin superfamily. This particular protein product is strongly expressed in the cochlea and the retina. Loss of function mutations have been reported to contribute to nonsyndromic progressive hearing loss.	Possibly progressing hearing impairment, need for regular checkups

## References

[1] Schleiermacher E, Schliebitz U, Steffens C (1974). Brother and sister with trisomy 10p: a new syndrome. *Humangenetik*.

[2] Granata P, Mazzola D, Righi R (2000). Molecular cytogenetics, RFLP analysis and clinical characterization of a *de novo* trisomy 10p case. *Annales de Genetique*.

[3] Schomig-Spingler M, Schmid M, Brosi W, Grimm T (1986). Chromosome 7 short arm deletion, 7p21→pter. *Human Genetics*.

[4] Hart DO, Santra MK, Raha T, Green MR (2009). Selective interaction between Trf3 and Taf3 required for early development and hematopoiesis. *Developmental Dynamics*.

[5] Deato MD, Tjian R (2008). An unexpected role of TAFs and TRFs in skeletal muscle differentiation: switching core promoter complexes. *Cold Spring Harbor Symposia on Quantitative Biology*.

[6] Hustinx ThWJ, Ter Haar BGA, Scheres JMJC, Rutten FJ (1974). Trisomy for the short arm of chromosome No. 10. *Clinical Genetics*.

[7] de Chieri P, Spatuzza E, Bonich JM (1978). Brother and sister with trisomy 10p. *Human Genetics*.

[8] Jalbert P, Sele B, Jalbert H (1980). Reciprocal translocations: a way to predict the mode of imbalanced segregation by pachytene-diagram drawing. *Genetics*.

[9] Sarfarazi M, Rezaie T (2003). Optineurin in primary open angle glaucoma. *Ophthalmology Clinics of North America*.

[10] Wiggs JL, Auguste J, Allingham RR (2003). Lack of association of mutations in optineurin with disease in patients with adult-onset primary open-angle glaucoma. *Archives of Ophthalmology*.

[11] Walsh T, Walsh V, Vreugde S (2002). From flies' eyes to our ears: mutations in a human class III myosin cause progressive nonsyndromic hearing loss DFNB30. *Proceedings of the National Academy of Sciences of the United States of America*.

[12] Simpson MA, Hsu R, Keir LS (2007). Mutations in FAM20C are associated with lethal osteosclerotic bone dysplasia (Raine syndrome), highlighting a crucial molecule in bone development. *American Journal of Human Genetics*.

[13] Simpson MA, Scheuerle A, Hurst J, Patton MA, Stewart H, Crosby AH (2009). Mutations in FAM20C also identified in non-lethal osteosclerotic bone dysplasia. *Clinical Genetics*.

[14] Fruttiger M, Karlsson L, Hall AC (1999). Defective oligodendrocyte development and severe hypomyelination in PDGF-A knockout mice. *Development*.

[15] Brisset S, Kasakyan S, L'Herminé AC (2006). *De novo* monosomy 9p24.3-pter and trisomy 17q24.3-qter characterised by microarray comparative genomic hybridisation in a fetus with an increased nuchal translucency. *Prenatal Diagnosis*.

